# Wearing-off Identification in Parkinson's Disease: The shapd-woq Study

**DOI:** 10.3389/fneur.2020.00116

**Published:** 2020-03-13

**Authors:** Ying Wan, Canxing Yuan, Xiaojun Hou, Wei Chen, ChunYan Wang, Shan Gao, Yuhui Wang, Lingjing Jin, Zhenguo Liu

**Affiliations:** ^1^Department of Neurology, Xinhua Hospital, Affiliated to Shanghai JiaoTong University, School of Medicine, Shanghai, China; ^2^Department of Neurology, Longhua Hospital Shanghai University of Traditional Chinese Medicine, Shanghai, China; ^3^Department of Neurology, The Second Military Medical University Affiliated Changhai Hospital, Shanghai, China; ^4^Department of Neurology, Shanghai Ninth People's Hospital, Shanghai Jiao Tong University School of Medicine, Shanghai, China; ^5^Department of Neurology, Shanghai Yangpu Hospital of Traditional Chinese Medicine, Shanghai, China; ^6^Department of Neurology, Shanghai JiaoTong University Affiliated the Sixth People Hospital, Shanghai, China; ^7^Department of Neurology, Shanghai Punan Hospital, Shanghai, China; ^8^Department of Neurology, Tongji Hospital, Tongji University School of Medicine, Shanghai, China

**Keywords:** Parkinson's disease, wearing-off phenomenon, patient self-assessments, pre-visiting screening, validity

## Abstract

**Objectives:** To clarify the frequency of wearing-off phenomenon (WO) and the validity of the Chinese version of the 9-item wearing-off questionnaire (CWOQ-9) in WO identification in this large population.

**Methods:** Parkinson's patients treated with antiparkinsonian medications were consecutively recruited into this observational, cross-sectional investigation. Patients completed the CWOQ-9 prior to the independent clinician assessment.

**Results:** A total of 1,385 patients were included in the analysis. The mean age was 69.7 ± 9.5 years and the mean disease duration was 5.8 ± 4.7 years. Clinicians identified WO in 763 patients, with an overall prevalence of 55.1%. In patients within 1 year of disease duration, clinicians diagnosed WO in eight patients, with a percentage of 12.9%. With the disease progression, the WO frequency gradually increased to 76.2% in patients with 10–15 years of disease duration. Then, it slowly decreased at a longer disease duration. The occurrence of WO was closely associated with the disease duration, H&Y staging, and levodopa daily dose. CWOQ-9 identified 1,071 patients (1071/1398, 77.33%) that had WO-related symptoms. The mean CWOQ-9 score was 3.4 ± 1.6. CWOQ-9 corresponded with clinician assessments of WO in 734 of 763 cases; clinicians disagreed with the CWOQ-9 considering the presence of WO in 337 of 1,071 cases. The sensitivity and specificity of CWOQ-9 were 96.2 and 45.8%, respectively.

**Conclusions:** WO occurred frequently at the early and middle stage of PD. CWOQ-9 was qualified as a pre-visiting screening tool for clinicians to better identify WO.

## Introduction

Parkinson's disease (PD) is a commonly seen, disabling neurodegenerative disease in aging people. With the aid of antiparkinsonian medications, PD patients could effectively control their motor symptoms and maintain a satisfactory living quality. As the disease progresses, the medication benefit would gradually decrease due to the occurrence of wearing-off phenomenon (WO). WO has been clearly defined as a predictable recurrence of Parkinson's symptoms that appears before the next scheduled dose and relieves after the anti-parkinsonian medications (1). However, most clinicians in China considered WO as a levodopa-related complication that usually occurs in the late stage of PD. WO was seldom screened in patients treated without levodopa.

WO was reported to strongly impair the health living quality of PD patients (2), but it could be amenable to treatment. Early identification is very important for the timely optimized medical intervention. In China, except for the movement disorder specialists in tertiary care hospitals, physicians in secondary care hospitals account for a large portion of the medical staff that provide medical service for PD patients (3). Clinicians' familiarity with the symptomatology of WO greatly influences his/her diagnosis of WO. Some other factors might influence the identification of WO, such as insufficient time and patients' misunderstanding (4). Some scales were developed to help clinicians to identify WO (5, 6). Among them, the 9-item wearing-off questionnaire (WOQ-9), a patient self-assessment scale, has been recommended by MDS as a WO diagnostic screening tool for its high sensitivity (7, 8). The Chinese version of WOQ-9 (CWOQ-9) was developed and validated in a small sample in Hong Kong (9). Currently, CWOQ-9 has not been widely used in clinical practice in mainland China. Here, we conducted this study with the purpose of investigating the prevalence of WO in this large population of PD patients. Moreover, we planned to clarify the sensitivity and specificity of CWOQ-9 vs clinician assessments in the identification of WO.

## Patients and Methods

### Study Design

This was a observational, cross-sectional study entitled Shanghai Parkinson disease WOQ study (shapd-woq study, clinical trail.gov ID: NCT03026595). From September 2017 to June 2018, the study consecutively recruited PD patients from the clinics the shapd-woq study group (Appendix 1), which was made up of 11 tertiary care hospitals and 27 secondary care hospitals from 15 districts of Shanghai. The inclusion criteria were as follows: (1) clinically established PD (10), (2) having received a stable anti-parkinsonian pharmacological treatment since the last clinical visit, (3) a clear clinical benefit during the medical treatment. The exclusion criteria were as follows: (1) clinically probable PD (10); (2) evidence of atypical, secondary, or hereditary parkinsonian syndromes; (3) a history of stroke, head trauma, head tumor, and head surgery; (4) a history of severe psychiatric diseases including depression and dementia; (5) unwillingness to complete this survey. All participants were willing to participate in the survey and signed the written informed consent form. The study was approved by the Research Ethics Committee of each center of the shapd-woq study group.

### The Definition of WO

In this study, WO was defined as a generally predictable reemergence of motor and non-motor symptoms that usually occurred before the next scheduled doses of antiparkinsonian medications and relieved after the doses, which occurs at least once a day (5, 6).

### Patient Self-Assessments

We used the Chinese version of the 9-item wearing-off questionnaire (CWOQ-9) as a patient self-assessment tool for WO. Each patient would complete the CWOQ-9 during the waiting time for the scheduled clinical visit, which was generally finished within 5 min. Assistance in reading or writing by the accompanying persons (usually spouse or offspring) was permitted. For each item, patients reported whether a symptom was present and whether it improved after the next dose of antiparkinsonian medication. If both were positive, one score was acquired. The patient would be considered as CWOQ-9 positive if he/she had at least one score in CWOQ-9.

### Clinician Assessments

Clinicians finished the assessments during the routine clinical visits. The assessments included a WO evaluation through history inquiries to patients and a case report form (CRF) recordation. In our study, the CRF contained demographic information, clinical characteristics [the time to motor symptom onset and Hoehn & Yahr (H&Y) staging], and medication information. Clinicians were blind to the patients' result of CWOQ-9 during the whole study. After the recruitment, they would complete a questionnaire in which some working information and reasons of diagnosing WO were recorded. All clinicians were trained for the WO definition and CRF recordation before the recruitment.

### Statistics

Descriptive statistics were used as required. Continuous variables were expressed as mean values and standard deviations. Categorical variables were noted as numbers and percentages. Mann–Whitney *U* test was used in the comparison of the continuous variables in abnormal distribution between two groups. χ^2^ test was used in the comparison of the categorical variables. Multivariate logistic regression models were used to identify the independent influencing factor of WO. Statistical significance was set at *p* < 0.05, with a two-tailed approach. Statistical computations were performed by SPSS.

## Results

### Characteristics of the Patients

A total of 1,504 PD patients were enrolled into the survey. Of them, 119 patients were excluded from the statistical analysis due to incomplete medication information (85) or incorrect CWOQ-9 filling (34). A total of 1,385 patients were finally included into the statistical analysis. 53.2% of the whole population were males, the mean age was 69.7 ± 9.5 years, the mean diagnosis duration was 5.8 ± 4.7 years, and the median H&Y staging was 2.0 ± 1.0. Most of the patients were treated with levodopa. The characteristics of the PD patients are shown in detail in Tables 1, 2.

**Table 1:** Baseline characteristics of the study population.

	**PD patients** **(*n* = 1,385)**	**Patients with WO (*n* = 763)**	**Patients without WO (*n* = 622)**	***p***
Male, *n* (%)^a^^,^^$^	737 (53.2%)	410 (53.7%)	327 (52.6%)	0.67
Age (years)^b^^,^^&^	69.7 (9.5)	70.1 (9.75)	69.1 (9.1)	0.05
Age at onset (years)^b^^,^^&^	64.0 (10.0)	63.2 (10.04)	65.0 (9.4)	0.001^*^
Weight (kg)^b^^,^^&^	62.2 (10.0)	61.7 (10.03)	62.8 (9.8)	0.035^*^
Disease duration (years)^b^^,^^&^	5.8 (4.7)	7.0 (4.8)	4.2 (4.1)	<0.001^*^
H&Y^b^^,^^&^	2 (1.0)	2.5 (1.0)	2.0 (1.0)	<0.001^*^
LD (mg/day)^b^^,^^&^	396.3 (242.7)	442.5 (248.9)	339.6 (222.3)	<0.001^*^
LED (mg/day)^b^^,^^&^	487.7 (272.7)	546.8 (280.9)	415.1 (243.5)	<0.001^*^
Duration of anti-parkinsonian medical treatment (years)^b^^,^^&^	3.9 (3.7)	4.8 (4.0)	2.89 (3.4)	<0.001^*^
Duration of levodopa treatment (years)^b^^,^^&^	3.7 (3.9)	4.5 (4.15)	2.7 (3.4)	<0.001^*^

*WO, Wearing-off; H&Y, Hoehn & Yahr; LD, levodopa dose; LED, levodopa equivalent dose*.

a *Presented as number (percentage)*.

b *Presented as mean (standard deviation)*.

$ *Data were analyzed by χ^2^ test*.

& *Data were analyzed by Mann–Whitney U test*.

* *p < 0.05 of the comparison between two groups*.

**Table 2:** Medication of the study population.

	**PD patients** **(*n* = 1,385)**	**Patients with WO (*n* = 763)**	**Patients without WO (*n* = 622)**	***p***
Levodopa-benserazide, *n*(%)^a^^,^^$^	1206 (87.1%)	680 (89.2%)	526 (84.6%)	0.010^*^
Levodopa-cabidopa, *n*(%)^a^^,^^$^	402 (29.0%)	275 (36.0%)	127 (20.4%)	<0.001^*^
Pramipexole, *n*(%)^a^^,^^$^	587 (42.4%)	344 (45.1%)	243 (39.1%)	0.024^*^
Piribedil, *n*(%)^a^^,^^$^	261 (18.8%)	147 (19.3%)	114 (18.3%)	0.657
Bromocriptine, *n*(%)^a^^,^^$^	5 (0.4%)	3 (0.4%)	2 (0.3%)	0.825
Rasagiline, *n*(%)^a^^,^^$^	22 (1.6%)	13 (1.7%)	9 (1.4%)	0.704
Selegiline, *n*(%)^a^^,^^$^	227 (16.4%)	136 (17.8%)	91 (14.6%)	0.110
Trihexyphenidyl	86 (6.2%)	50 (6.6%)	36 (5.8%)	0.557
Amantadine, *n*(%)^a^^,^^$^	128 (9.2%)	89 (11.7%)	39 (6.3%)	0.001^*^
Entacapone, *n*(%)^a^^,^^$^	15 (1.1%)	10 (1.3%)	5 (0.8%)	0.440
Numbers of medications	2 (1, 3)	2 (2, 3)	2 (1, 2)	<0.001^*^
**Medical treatment strategies, *n*(%)^a^^,^^$^**				<0.001^*^
Levodopa monotherapy	390 (28.2%)	185 (24.2%)	205 (33.0%)	
Levodopa plus other drugs	902 (65.1%)	541 (70.9%)	361 (58.0%)	
DA monotherapy	77 (5.6%)	28 (3.7%)	49 (7.9%)	
Non-dopaminergic treatment	16 (1.2%)	9 (1.2%)	7 (1.1%)	

*WO, Wearing-off; DA, dopamine agonist*.

a *Presented as number(percentage)*.

$ *Data were analyzed by χ^2^ test*.

* *p < 0.05 of the comparison between two groups*.

### Characteristics of the Clinicians

Fifty-one clinicians participated in this study. Among them, 20 clinicians worked in tertiary care hospitals and 31 clinicians worked in secondary care hospitals. Most of the clinicians majored in neurology (47/51), and four clinicians majored in traditional Chinese medicine. For the clinicians, the mean years in practice in the field of PD was 9.4 ± 7.8 and the mean number of PD patients that they served per week was 14.7 ± 14.0.

### Patient Self-Assessments of WO

**Figure 1:**
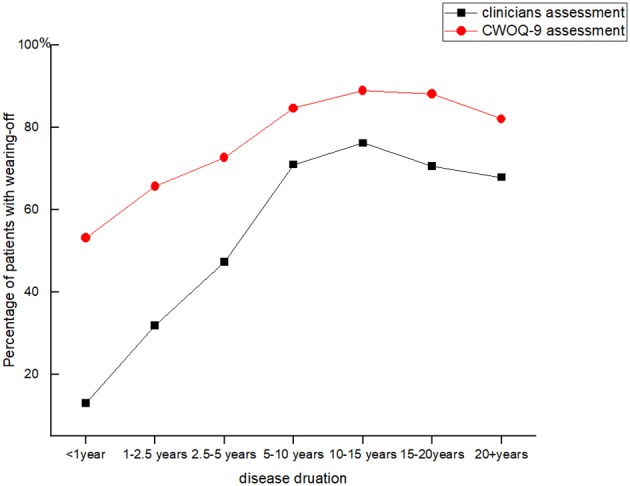
Two different methods identified Wearing-off in Parkinson's patients of different disease durations.

CWOQ-9 identified 1,071 patients (1071/1398, 77.3%) as having symptom fluctuations (Figure 1). The mean CWOQ-9 score was (3.4 ± 1.6). 60.4% (647/1071) of patients experienced pure motor fluctuations, 38.3% (410/1071) of patients experienced both motor and non-motor fluctuations, whereas 1.3% (14/1071) experienced only non-motor fluctuations. The most common motor symptom was movement slowness and the most common non-motor symptom was pain/aching.

### Clinician Assessments of WO

Clinicians identified WO in 763 patients, with the overall prevalence of WO being 55.1%. Most of the patients with WO were treated with levodopa, whereas 37 patients received dopamine agonists or non-dopaminergic medications. Further analysis showed that the WO frequency varied among different disease durations. WO was identified in 12.9% (8/62) of patients within 1 year of disease duration. The percentage of patients with WO continuously increased with the disease progression. It reached the highest value of 76.2% (160/210) in patients with 10–15 years of disease duration. Then, it gradually dropped to 67.9% (19/28) in patients with over 20 years of duration (Figure 1).

In terms of WO evaluation, the most frequently used question was symptom response to medications, with a frequency of 100%. It was followed by the fluctuating features of motor symptoms (98%), timing of symptom response to medications (72.5%), symptom fluctuation occurring at a fixed time per day, which lasts for days (54.9%), and fluctuating features of non-motor symptoms (52.90%). In addition, 56.9% of the clinicians considered that the presence of motor fluctuation was required for WO diagnosis.

### Characteristics of Patients With WO

According to clinician assessments, patients were divided into the WO group and Non-WO group. Compared with the Non-WO group, the WO group showed younger age at onset (*p* = 0.001), lower weight (*p* = 0.035), longer disease duration (*p* < 0.001), higher H&Y staging (*p* < 0.001), higher daily levodopa dose (LD) (*p* < 0.001) and daily levodopa equivalent dose (LED) (*p* < 0.001), and longer duration of antiparkinsonian medical treatment (*p* < 0.001) and levodopa treatment (*p* < 0.001). In terms of the medication types, the WO group used levodopa/benserazide (*p* = 0.010), levodopa/cabidopa (*p* < 0.001), pramipexole (*p* = 0.024), and amantadine (*p* = 0.010) more frequently than the Non-WO group. In addition, the WO group was much frequently treated with polytherapy of levodopa and other medications compared to the Non-WO group (70.9 vs. 58.0%, *p* < 0.001). No difference was found in the gender ratio or age between the two groups. In order to identify the WO-associated factors, we included all factors that showed significant differences into the multivariate logistic regression model. It showed that disease duration (OR = 1.101, 95% CI = 1.063–1.141, *p* < 0.001), H&Y staging (OR = 1.717, 95% CI = 1.452–2.030, *p* < 0.001), and the LED (OR = 1.001, 95% CI = 1.001–1.002, *p* < 0.001) were associated with the occurrence of WO.

### Comparison Between Clinician Assessments and Patient Self-Assessments

CWOQ-9 corresponded with clinician identification of WO in 734 of 763 patients; clinicians disagreed with the CWOQ-9 considering the presence of WO in 337 of 1,071 cases. Considering the clinician identification as a gold standard, the sensitivity of CWOQ-9 was 96.2% (95% CI = 94.5–97.4%), the specificity of CWOQ-9 was 45.8% (95% CI = 41.9–49.8%). We further compared the patients of clinician identification (–) and CWOQ-9 (+) to the patients of clinicians identification (+) and CWOQ-9 (+). Results showed that patients of clinician identification (+) and CWOQ-9 (+) were characterized by a younger age at onset (*p* = 0.018), a longer disease duration (*p* < 0.001), a higher H&Y staging (*p* < 0.001), a higher CWOQ-9 score, and higher frequencies in most of the motor and non-motor symptoms, compared with patients of clinician identification (–) and CWOQ-9(+) (Table 3).

**Table 3:** Comparison of the characteristics between WO patients of different assessment methods.

	**Clinician identification (+) and CWOQ-9 (+) (*n* = 734)**	**Clinician identification (–) and CWOQ-9 (+) (*n* = 337)**	***p***
Male, *n*(%)^a^^,^^$^	395 (53.8%)	175 (51.9%)	0.57
Age (years)^b^^,^^&^	70.0 (9.8)	69.3 (9.5)	0.264
Age at onset (years)^b^^,^^&^	63.0 (10.5)	64.9 (9.8)	0.018^*^
Disease duration (years)^b^^,^^&^	7.0 (4.8)	4.6 (4.3)	<0.001^*^
H&Y^b^^,^^&^	2.5 (0.9)	2.0 (0.8)	<0.001^*^
CWOQ-9 score^b^^,^^&^	3.7 (1.6)	2.7 (1.6)	<0.001^*^
Motor symptoms			
Tremor^a^^,^^$^	533 (72.6%)	240 (71.2%)	0.635
Slowness of movement^a^^,^^$^	639 (87.1%)	217 (64.4%)	<0.001^*^
Stiffness^a^^,^^$^	478 (65.1%)	146 (43.3%)	<0.001^*^
Reduced dexterity^a^^,^^$^	449 (61.2%)	112 (33.2%)	<0.001^*^
Muscle cramping^a^^,^^$^	123 (16.8%)	34 (10.1%)	0.004
Non-motor symptoms			
Mood changes^a^^,^^$^	162 (22.1%)	44 (13.1%)	0.001^*^
Pain/aching^a^^,^^$^	191 (26.0%)	48 (14.2%)	<0.001^*^
Cloudy mind/ slowness of thinking^a^^,^^$^	80 (10.9%)	29 (8.6%)	0.277
Anxiety/panic attacks^a^^,^^$^	48 (6.5%)	30 (8.9%)	0.166

WO, Wearing-Off; CWOQ-9, Chinese version of 9-item of wearing-off questionnaire;

a *Presented as number(percentage)*.

b *Presented as mean (standard error)*.

$ *Data were analyzed by χ2test*.

& *Data were analyzed by Mann-Whitney U test*.

* *p < 0.05 of the comparison between two groups*.

## Discussion

This is a large population-based investigation of WO in Chinese patients with PD. The data indicated that WO was very common as 77.3% of the population were CWOQ-9 positive and 55.1% of the population were diagnosed by clinicians as having WO. The overall prevalence of WO was consistent to the DEEP study (2) and a recent multi-center survey in Japan (11). It was a bit higher than the previous multi-center survey in China (12). This might be partly attributed to the difference in the criteria of WO diagnosis and patient recruitment between the two studies. We deleted the requirements of the use of levodopa and the benefit duration of a given levodopa dose in the inclusion criteria. In our study, only 6.6% of our study population were not treated with levodopa (mainly dopamine agonists); however, over one third (37/93) of them were identified by clinicians to have WO, which was slightly less than a previous study (13). In the past years, clinicians seldom inquired of patients treated without levodopa about WO-related symptoms in the clinical visit. Our results indicated that WO would also be required to be screened in patients treated with medications other than levodopa.

Our study found that WO frequently occurred at the early and middle stage of PD as clinicians identified WO in 12.9% of patients within 1 year of disease duration. The frequency increased as the disease progressed; however, it presented with a decreased tendency in patients with over 15 years of disease duration. These patients were always at the late stage of PD and poorly responded to levodopa (13, 14). During the levodopa challenge test, the late-stage patients acquired a slight improvement in the UPDRS motor scores by 11.35%; meanwhile, they experienced serious dyskinesia and a series of adverse effects (15). An observation study reported that late-stage PD patients were generally undertreated and seldom felt a significant improvement after a given levodopa dosage (16). Therefore, patients at very late stage experienced less frequent motor or non-motor fluctuations.

Previous studies reported that 10% of patients per year experienced motor fluctuations (mainly WO) after the levodopa therapy, and levodopa treatment duration was a main cause of developing WO (17). Accordingly, in the past years, “levodopa phobia” induced both clinicians and patients to delay the levodopa treatment as long as possible. Our data found that WO was not associated with levodopa treatment duration, which was in accordance with a prospective observational study (18). Further, both Zhang's study and the STRID-PD study proposed that controlling LD under 400 mg would effectively lower patients' risk of developing WO (19, 20). Accordingly, patients might obtain more medical benefit through the polytherapy strategy of levodopa and other medications than delayed initiation of levodopa treatment.

Our study supported the fact that CWOQ-9 was a very simple and sensitive screening scale. Most of the patients finished the CWOQ-9 within several minutes. Nearly all of PD patients diagnosed with WO by clinicians were identified by CWOQ-9 as having WO. However, the CWOQ-9 had a very low specificity as clinicians disagreed with the CWOQ-9 considering the presence of WO in 337 of 1,071 cases. Interestingly, the specificity of some other patient self-assessment questionnaires was not as high as its sensitivity (2, 13). The low specificity of CWOQ-9 might be attributed to several factors. On one hand, CWOQ-9 only captured the core feature of WO, which is symptom response to medications. In contrast, clinicians considered more other features when they diagnosed patients with WO, including fluctuating features of motor symptoms (98%), timing of symptom response to medications (72.5%), symptom fluctuation occurring at a fixed time per day, which lasts for days (54.9%), and fluctuating features of non-motor symptoms (52.90%). Further analysis found that PD patients with WO identified by both clinicians and CWOQ-9 were featured by longer disease duration, higher H&Y staging, as well as more fluctuating motor and non-motor symptoms, compared with PD patients that clinicians disagreed with the CWOQ considering the presence of WO. We speculated that WO-related symptoms might be more distinct in clinical features in these patients, which was highly consistent to the information used in the clinicians' diagnosis of WO. On the other hand, our study found that over half of clinicians considered the presence of fluctuating motor symptoms as a requirement for WO diagnosis. Therefore, patients would not be diagnosed as having WO if they were experiencing pure non-motor fluctuations, though they accounted for a very small percentage of our study population. Besides, compared with clinician assessments, CWOQ-9 exhibited a detailed list of many commonly seen motor and non-motor symptoms and clear instructions, which facilitates patients a better identification of WO experience. There was the possibility that clinicians underestimated the prevalence of WO in the clinical visits. Therefore, we suggested a popularization of pre-visiting screening for WO in clinical practice.

There are some limitations in our study. The study did not include PD patients who were unable to go to the hospitals due to serious motor disability. It might influence WO prevalence. In addition, we evaluated the motor function through H&Y staging. It could not provide a very detailed information on motor symptoms. However, H&Y staging was quite suitable for the large population investigation with many evaluators because of its simple operation and high consistency.

## Conclusion

Our study supported the fact that WO occurred frequently at the early and middle stage of PD. With the disease progression, WO might vary in the clinical manifestations. CWOQ-9 was a very sensitive screening scale and could be used in pre-visiting screening for WO to help clinicians to more effectively identify WO during a limited time.

## Data Availability Statement

The datasets generated for this study are available on request to the corresponding author.

## Ethics Statement

The studies involving human participants were reviewed and approved by Research ethics Committees of Xinhua Hospital affiliated to Shanghai Jiaotong University School of Medicine. The patients/participants provided their written informed consent to participate in this study.

## Author Contributions

LJ and ZL designed the original study. YWan wrote the manuscript. XH, WC, SG, CW, and YWang input the data. CY analyzed the data. The members of the shapd-woq study group recruited the patients. ZL revised the manuscript. ZL and LJ supervised the study. All authors read and approved the final manuscript.

### Conflict of Interest

The authors declare that the research was conducted in the absence of any commercial or financial relationships that could be construed as a potential conflict of interest.
