# Supplementary material for: Wearing-off Identification in Parkinson's Disease: The shapd-woq Study
Source: Front Neurol. 2020 Mar 13;11:116. doi: 10.3389/fneur.2020.00116 (PMC7083219; doi:10.3389/fneur.2020.00116)
Supplement: Supplementary file 1 [file Data_Sheet_1.docx]

The shapd-woq study Group Members

| Ying Wan | Department of Neurology, Xinhua Hospital, Affiliated to Shanghai JiaoTong University, School of Medicine | Shanghai, China |  |
| --- | --- | --- | --- |
| Zhenguo Liu | Department of Neurology, Xinhua Hospital, Affiliated to Shanghai JiaoTong University, School of Medicine | Shanghai, China |  |
| Canxing Yuan | Department of Neurology, Longhua Hospital Shanghai University of Traditional Chinese Medicine | Shanghai, China |  |
| Lingjin Jin | Department of Neurology, Tongji Hospital, Tongji University school of Medicine | Shanghai, China |  |
| Xiaojun Hou | Department of Neurology, The Second Military Medical University affiliated Changhai Hospital | Shanghai, China |  |
| Wei Chen | Department of Neurology, Shanghai Ninth People’s Hospital, Shanghai Jiao Tong University School of Medicine | Shanghai, China |  |
| Chunyan Wang | Department of Neurology, Shanghai Yangpu Hospital of Traditional Chinese Medicine | Shanghai, China |  |
| Shan Gao | Department of Neurology, Shanghai JiaoTong University affiliated the Sixth People Hospital, South Campus | Shanghai, China |  |
| Yuhui Wang | Department of Neurology, Shanghai Punan Hospital | Shanghai, China |  |
| Ying Guan | Department of Neurology, Renhe Hospital, Baoshan District | Shanghai, China |  |
| Wenzhao Wang | Department of Neurology, Shanghai Changzheng Hospital | Shanghai, China |  |
| Dongya Huang | Department of Neurology, Shanghai East Hospital, Tongji University | Shanghai, China |  |
| Xinyi Wang | Department of Neurology, Shanghai East Hospital, Tongji University | Shanghai, China |  |
| Feng Yu | Department of Neurology, Jiangwan hospital, Hong kou district | Shanghai, China |  |
| Wen Li, | Department of Neurology, Kong Jiang Hospital of Yang pu district | Shanghai, China |  |
| WenTao Li | Department of Neurology, Shanghai Municipal Hospital of Traditional Chinese Medicine,Shanghai University of Traditional Chinese Medicine | Shanghai, China |  |
| Xiaohui Zhao | Department of Neurology, Shanghai Pudong New Area People’s Hospital | Shanghai, China |  |
| Yong Bi | Department of Neurology, Shanghai Fourth People’s Hospital Affiliated to Tongji University School of Medicine | Shanghai, China |  |
| Weidong Pan | Department of Neurology, ShuGuang Hospital Affiliated to Shanghai University of Traditional Chinese Medicine | Shanghai, China |  |
| Yi Zhao | Department of Neurology, Xuhui Center Hospital in Shanghai | Shanghai, China |  |
| YingChun Zhao | Department of Neurology, Shanghai Songjiang District Central Hospital | Shanghai, China |  |
| Changde Wang | Department of Neurology, Shanghai TCM-Integrated hospital affiliated to Shanghai University of Traditional Chinese Medicine | Shanghai, China |  |
| LongXuan Li | Department of Neurology, Gongli Hospital, Pu Dong New Area | Shanghai, China |  |
| Qing Dong | Department of Neurology, Renji Hospital, South Campus, Affiliated to Shanghai Jiaotong University, School of Medicine | Shanghai, China |  |
| De Shi | Department of Neurology, Xinhua hospital Affiliated to Shanghai Jiaotong University School of Medicine, Chongming Branch | Shanghai, China |  |
| Fusheng Niu | Department of neurology, Shanghai Public Health Clinical Center | Shanghai, China | |
| QiuDong Wang | Department of neurology, Pudong New Area Hospital of Traditional Chinese Medicine | Shanghai, China | |
| Ying Wang | Department of Neurology, Ruijin Hospital, Affiliated to Shanghai Jiaotong University School of Medicine | Shanghai, China | |
| Lihong Huang | Department of Neurology, Zhabei Center hospital, Jiang’an District | Shanghai, China | |
| Biaoji Zhao | Department of Neurology, Shanghai Beizhan Hospital | Shanghai, China | |
| GuoYi Li | Department of Neurology, Shanghai Putuo district central hospital | Shanghai, China | |
| Weiwen Wu | Department of Neurology, Zhongshan Hospital Qingpu Branch, Fudan University | Shanghai, China | |
| ChaoRong Zhao | Department of TCM , Shanghai Putuo District Hospital of Traditional Chinese Medicine | Shanghai, China | |
| Hui Wang | Department of Neurology, Dahua Hospital of Xu hui District | Shanghai, China | |
| Mengyuan Qu | Department of Neurology, Shanghai Baoshan District Combining Traditional Chinese and Western Medicine Hospital | Shanghai, China | |
| Jing Zhao | Department of Neurology, Minhang Center hospital, Fudan University | Shanghai, China | |
| Ping Ding | Department of Neurology, Huadong Hospital Affiliated to Fudan University | Shanghai, China | |
| Yunhua Yue | Department of Neurology, Yangpu hospital, Tongji University School of Medicine | Shanghai, China | |
| Qi Zhong | Department of Neurology, Huangpu Center hospital | Shanghai, China | |
| Yang Zhou | Department of Neurology, Yueyang Hospital of intergrated Traditional Chinese and Western Medicine, Shanghai University of Traditional Chinese Medicine | Shanghai, China | |
